# Vestibular function in cases of posterior semicircular canal canalolithiasis and cupulolithiasis

**DOI:** 10.3389/fneur.2024.1369193

**Published:** 2024-02-29

**Authors:** Xu Wenyan, Yue Lifeng, Wu Jing, Jiang Hui

**Affiliations:** ^1^Department of Neurology, Dongzhimen Hospital, Beijing University of Chinese Medicine, Beijing, China; ^2^Department of Otolaryngology, Dongfang Hospital, Beijing University of Chinese Medicine, Beijing, China

**Keywords:** benign paroxysmal positional vertigo, video head impulse test, vestibular evoked myogenic potentials, cupulolithiasis, vestibular function

## Abstract

**Objective:**

To analyze and compare the vestibular function of posterior canal cupulolithiasis and canalolithiasis.

**Methods:**

The results of posterior cupulolithiasis in 45 cases, posterior canalolithiasis in 122 cases and 19 healthy controls were analyzed retrospectively.

**Results:**

The abnormal rates of vHIT in the canalolithiasis group and the cupulolithiasis group were 42.6 and 37.8%, respectively, both higher than those in the control group (both *p* < 0.05); there was no statistically significant difference between two BPPV groups (*p* = 0.573). The abnormal vHIT in 76.9% of the canalolithiasis cases and 82.4% of the cupulolithiasis cases showed normal gain with saccades, with no difference between the groups (*p* = 0.859). The lesion location of vHIT in the two groups did not show a correlation with the affected side of BPPV (both *p* > 0.05). 84.4% of canalolithiasis and 65.0% of cupulolithiasis had abnormal VEMP results, with no significant differences in abnormality rates or sides (both *p* > 0.05). Abnormal results of VEMPs did not show any correlation with side (*p* > 0.05). The results of pc-ca and pc-cu were both abnormal in 14 cases and 7 cases, and there was no correlation between the site and side of the injury (all *p* > 0.05).

**Conclusion:**

The results of vHIT and VEMP in pc-cu and pc-ca were partially abnormal, but they did not show any correlation with side of BPPV. It can be considered that there are scattered vestibular peripheral organ damage in both groups.

## Introduction

Benign paroxysmal positional vertigo (BPPV) is the most common vestibular disease (1), and its pathogenesis is related to the detachment of otolith particles from the macula of utricle. Canalolithiasis and cupulolithiasis are widely accepted theories at present. Posterior canal-canalolithiasis (pc-ca) is the most common type of BPPV; posterior canal-cupulolithiasis (pc-cu) is less common, and its characteristic nystagmus is a persistent nystagmus with rotational and vertical components induced at the half hallpike (HH) position (2, 3).

Previous studies have shown a correlation between BPPV and vestibular dysfunction (4, 5). The purpose of this study was to explore whether there are differences in vestibular function between pc-ca and pc-cu and the association of the injured side. This study conducted a retrospective analysis of the results of video head impulse test (vHIT) and vestibular evoked myogenic potentials (VEMPs) for these two types of BPPV, as reported below.

## Materials and methods

### Sample sources

This study was a single-center retrospective study. The cases were from patients with posterior semicircular canal BPPV who were treated in Tongzhou Branch of Dongzhimen Hospital Affiliated to Beijing University of Chinese Medicine from September 2021 to December 2022; the control group was composed of 19 healthy employees of the hospital. This study was approved by the Medical Ethics Committee (2023DZMEC-188).

### Inclusion and exclusion criteria

Inclusion criteria were: (1) The diagnostic criteria were based on the BPPV diagnostic criteria published by the Bárány Society (2), and patients considered as unilateral PC-CA and successfully treated with manual reduction on the same day were included in the pc-ca group.

Inclusion criteria of pc-cu group: (a) Recurrent attacks of positional vertigo or positional dizziness provoked by lying down or turning over in the supine position. (b) Positional nystagmus elicited after a brief or no latency by a “half Dix-Hallpike maneuver” on the affected side, beating torsionally with the upper pole of the eye to the lower ear and vertically upward (to the forehead) and lasting >1 min. (c) The nystagmus reversal with head inversion (3). (d) Not attributable to another disorder. (3) There was no limitation on gender, and the age was 18–80 years old. (4) Video nystagmography (VNG), vHIT, and/or VEMPs examinations were completed, and the examinations were completed on the same day as the diagnosis and reduction of otolith.

Exclusion criteria were: (1) Alcohol intake within 48 h. (2) Use of vestibular inhibitors within 72 h. (3) Spontaneous nystagmus is present. (4) Comorbidity with the following diseases: vestibular neuritis, Meniere’s disease, sudden deafness, acoustic neuroma, vestibular migraine. (5) Multiple canals BPPV or traumatic secondary BPPV.

### Examination methods

The diagnosis of BPPV was made using a video nystagmus electrogram (Otometrics ICS Chartr 200), where positional nystagmus was observed and recorded under fixation extinction. All cases completed Dix-hallpike and Roll position tests; for those considered for a diagnosis of pc-cu, HH position (3) and nose down (ND) position tests were added.

All cases were treated with Epley maneuver immediately after diagnosis. After reduction, the Dix-Hallpike test was retested on the affected side. The disappearance of positional nystagmus and vertigo symptoms was considered as successful reduction.

vHIT detection is performed using a video head impulse transducer (Otometrics ICS Impulse). At least 15 effective head-implulse events were recorded for each semicircular canal. Both head and eye movement curves were recorded; and the gain value, gain asymmetry (GA), peak saccade velocity, corrective saccades (CS). GA = [1-lower gain/higher gain] × 100%. The ratio of CS = [the number of head impulses with CS]/[trials number] × 100%. The following conditions are considered abnormal in vHIT: (1) horizontal semicircular gain <0.8, vertical semicircular gain <0.7 (6), accompanied by CS. (2) normal gain, with CS in more than 50% of trials, i.e., the ratio of CS is over 50% (7, 8).

The VEMPs were detected using an auditory evoked potential instrument (Otometrics Chartr EP200). An in-ear air-conduction headphone was used with a 500 Hz short pure tone stimulus, giving sound intensity of 95/97dBnHL and a frequency of 5.1 beats/s. The typical waveform of cVEMP is a trough around 13 ms (recorded as p13) and a peak around 23 ms (recorded as n23); the typical waveform of oVEMP is a peak around 10 ms (recorded as n10) and a peak around 15 ms (recorded as p15). Calculate the amplitude and asymmetric ratio (AR), AR = (|right side amplitude-left side amplitude|)/(right side amplitude+left side amplitude) × 100%. The detection of each part was repeated 2–3 times. The repeatable waveform was used for subsequent calculations. If no repeated waveform was found after 3 times of stimulation, the vemp at that location was determined to be absent. Normal data for VEMPs were obtained based on healthy controls. Abnormal VEMPs were defined with any of the three criteria, i.e., absent responses, asymmetric responses (AR N mean + 2 standard deviation [SD]) or delayed latencies (latency N mean + 2 SD) (9).

### Statistical methods

The comparison of mean and distribution was conducted using SPSS 26.0 software. Count data were expressed as n/% and compared using the chi-square test. Normal measurement data were expressed as mean ± standard deviation and compared using the *t* test. Non-normal data were expressed as Q50 (Q25, Q75) and compared using the Mann–Whitney U test between two independent samples. The paired samples were compared using the Wilcoxon signed rank test. The Kruskal-Wallis test was used for comparison among three groups. Bonferroni correction was used for pairwise comparisons. *P* < 0.05 was considered statistically significant. The correlation analysis was conducted by employing ROC curves with the aid of MedCalc 22 software.

## Results

### General information

A total of 122 cases in the pc-ca group, 45 cases in the pc-cu group, and 19 cases in the control group were included. There were no statistically significant differences in gender, age, and BPPV affected side among the groups (all *p* > 0.05, Table 1). According to the order of reduction treatment and vHIT examination, the pc-ca group was divided into two subgroups: before reduction (21 cases) and after reduction subgroup (101 cases). The before reduction-subgroup completed the vHIT examination before performing the repositioning maneuvers. The vHIT examination was performed after repositioning maneuvers in after reduction-subgroup.

**Table 1:** General information.

	pc-ca	pc-cu	Control	χ^2^	*p*-value
Total	122	45	19		
Sex (M/F)	36:86	10:35	7:12	1.572	0.456
Age (year)	54.79 ± 14.80	54.16 ± 14.25	51.16 ± 14.05	0.984	0.611
Affected side (R/L)	78:44	22:23		3.098	0.109

All cases received Epley maneuver reduction on the day of examination. No significant change in positional nystagmus was observed in all cases of pc-cu after Epley maneuver reduction. 24/45 (53.3%) cases of pc-cu had a 14-day outpatient follow-up record and completed at least bilateral Dix-Hallpike, HH and roll tests, showing disappearance of positional nystagmus and/or relief of vertigo symptoms. No cases of pc-cu converted to typical pc-ca or other types of BPPV.

### vHIT results

#### Comparison of abnormality detection

A total of 69 (41.3%) cases of BPPV had abnormal vHIT, including 52 (42.6%) cases in the pc-ca group and 17 (37.8%) cases in the pc-cu group. The abnormal rates of vHIT in pc-ca group and pc-cu group were significantly different from those in the control group (*χ*^2^ = 12.83, *p* = 0.000; *χ*^2^ = 9.774, *p* = 0.002, respectively). There was no significant difference in the abnormal rate of vHIT between the pc-ca and pc-cu groups (*χ*^2^ = 0.318, *p* = 0.573). The rates of left-sided abnormalities were 26.9 and 25.3%, respectively; and the rates of bilateral abnormalities were 30.8 and 29.4%, respectively, with no statistically significant differences between the two groups (all *p* > 0.05, Table 2).

**Table 2:** Comparison of abnormal vHIT detection.

	pc-ca	pc-cu	Control	*χ* ^2^	*p*-value
Abnormal/normal	52:70	17:28	0:19	12.742	0.002
Low/normal gain	12:40	3:14		0.018	0.895
Affected side	22 (42.3%)	6 (35.3%)		0.261	0.609
Healthy side	14 (26.9%)	6 (35.3%)		0.436	0.509
Bilateral	16 (30.8%)	5 (29.4%)		0.011	0.916
Responsibility PC	9 (17.3%)	2 (11.8%)		0.026	0.873

In the pc-ca group, the abnormal rates of vHIT in the subgroups after reduction and before reduction were 45.5% (46/101) and 28.5% (6/21), respectively, with no statistically significant difference between the subgroups (*χ*^2^ = 2.048, *p* = 0.152). In the abnormal vHIT cases, 40 (76.9%) cases of pc-ca and 14 (82.4%) cases of pc-cu showed normal gain with saccades, with no difference between the groups (*χ*^2^ = 0.018, *p* = 0.895). ROC curve analysis showed that the abnormal semicircular canal and side of vHIT were not correlated with the responsible semicircular canal of BPPV (all *p* > 0.05).

#### Comparison of gain, GA, and CS

The average gains of each semicircular canal in the three groups of vHIT were all within the normal range (Table 3). The horizontal semicircular canal gain in the pc-ca group was lower than that in the pc-cu group and the control group (Bonferroni correction *p* < 0.05). There were no statistically significant differences in GA values between the synergistic semicircular canals in each group (all *p* > 0.05). In the pc-ca group, there were no statistically significant differences in the average and healthy side and affected side gains of each canal between the subgroups before and after reduction (all *p* > 0.05).

**Table 3:** Comparison of gain values between groups.

		pc-ca	pc-cu	Control	*χ* ^2^	*p*-value
Mean value	Healthy side	0.86 (0.80, 0.93)	0.87 (0.81. 1.00)	0.91 (0.84, 0.95)	4.004	0.135
	Affected side	0.87 (0.80, 0.93)	0.86 (0.80, 0.98)	0.91 (0.84, 0.95)	2.266	0.322
AC	Healthy side	0.78 (0.67, 0.90)	0.81 (0.65, 0.91)	0.82 (0.68, 0.84)	1.019	0.601
	Affected side	0.83 (0.67, 0.97)	0.83 (0.67, 0.97)	0.82 (0.68, 0.84)	1.804	0.406
HC	Healthy side	1.04 (0.96, 1.13)	1.08 (1.01, 1.19)	1.12 (1.04, 1.18)	8.580	0.014
	Affected side	1.03 (0.95, 1.13)	1.11 (1.00, 1.20)	1.12 (1.04, 1.18)	10.360	0.006
PC	Healthy side	0.79 (0.70, 0.89)	0.83 (0.70, 0.97)	0.83 (0.73, 0.91)	1.930	0.381
	Affected side	0.77 (0.66, 0.85)	0.76 (0.66, 0.89)	0.83 (0.73, 0.91)	3.372	0.185

There were no statistically significant differences in the average percentages of healthy side and affected side saccades, as well as the percentage of vertical semicircular canal saccades, among the three groups (all *p* > 0.05). The proportion of average horizontal semicircular saccades in the pc-ca group was higher than that in the control group (standard test statistic = 3.299, Bonferroni correction *p* = 0.003), but there was no difference in the proportion of horizontal semicircular saccades between the affected side and the healthy side within the group (Wilcoxon test, *Z* = −0.588, *p* = 0.557) (Table 4).

**Table 4:** Comparison of the CS (% of tirals) between groups.

	pc-ca	pc-cu	control	*χ* ^2^	*p*-value
Affected side	11.70 (4.30, 24.01)	11.33 (4.50, 22.17)	5.67 (4.33, 9.33)	5.33	0.070
Healthy side	10.00 (4.00, 22.65)	11.00 (2.33, 22.50)	5.67 (4.33, 9.33)	2.43	0.297
Ac average	3.00 (0.00, 7.00)	3.00 (0.00, 6.75)	7.00 (0.00, 10.50)	5.194	0.074
Hc average	18.5 (6.00, 52.63)	14.0 (3.00, 39.25)	6.50 (2.50, 10.50)	11.362	0.003
Pc average	3.50 (0.00, 13.00)	8.00 (1.50, 22.00)	3.50 (3.30, 9.50)	2.816	0.245

### VEMP results

#### Comparison of abnormality detection

The normal values for VEMP were established using healthy controls. The normal values for oVEMP-AR and cVEMP-AR were set at less than 30.29 and 29.11%, respectively. Additionally, the reference values for latency parameters were defined as follows: P13 < 14.16 ms, N23 < 25.33 ms, N10 < 11.44 ms, and P15 < 18.10 ms.

Thirty-two cases in pc-ca group and 20 cases in pc-cu group completed VEMP examination.

In the pc-ca group, 27 (84.4%) cases exhibited abnormal VEMP results, including 17 cases with abnormal cVEMP, 23 cases with abnormal oVEMP, and 13 cases with abnormalities in both results. In the pc-cu group, 16 (65.0%) cases exhibited abnormal VEMP results, including 12 cases with abnormal cVEMP, 12 cases with abnormal oVEMP, and 8 cases with abnormalities in both results. In the control group, there were 6 (31.6%) cases of abnormal VEMP, including 4 cases of abnormal cVEMP and 2 cases of abnormal oVEMP, and there was no case with abnormalities in both results. There was no statistically significant difference in the abnormal rate of VEMP between the pc-ca group and the pc-cu group (both *p* > 0.05); however, there were significant differences when compared to the control group (both *p* < 0.05).

Neither cVEMP nor oVEMP in two groups showed correlation with the affected side of BPPV (both *p* > 0.05).

The specific abnormal items of the two groups are shown in Table 5. Among them, delayed latencies were most commonly observed in P13 and N10 (46.2 and 28.8%, respectively). There was no statistically significant difference in the distribution of each abnormality between the two groups (all *p* > 0.05).

**Table 5:** Comparison of abnormalities between groups.

		AR abnormality		Absence		Delayed latency			
		cVEMP	oVEMP	cVEMP	oVEMP	P13	N23	N10	P15
pc-ca *n* = 32	Affected/Unaffected side	0:6	2:3	2:2	6:7	9:9	1:0	9:11	2:3
	Total*	6	5	4	7	14	1	11	3
pc-cu *n* = 20	Affected/Unaffected side	3:4	3:1	0:1	5:3	7:3	1:1	3:2	0:0
	Total	7	4	1	5	10	2	4	0
Control *n* = 19	Total	1	0	0	0	2	1	2	0

*Bilateral abnormalities were counted as 1 case in the total.

#### Comparison of the latency and amplitude between groups

Four (12.5%) cases in the pc-ca group had cVEMP absence, and 7 (21.9%) had oVEMP absence. In the pc-cu group, 1 patient (5.0%) had cVEMP absence, and 5 (25.0%) had oVEMP absence. The control group exhibited no absence of VEMPs. After excluding cases with VEMP absence, the average latency, amplitude, and AR were compared between groups (Table 6).

**Table 6:** Comparison of latency, amplitude, and asymmetry ratio between groups.

		pc-ca	pc-cu	Control	*χ* ^2^	P值
P13 (ms)	Affected side	13.15 (12.35, 14.70)	16.65 (12.40, 14.86)	12.30 (12.20, 13.55)	4.677	0.096
	Healthy side	12.90 (12.10, 14.58)	12.75 (12.17, 13.60)	12.30 (12.20, 13.55)	1.433	0.488
N23 (ms)	Affected side	21.23 (20.30, 22.80)	22.30 (20.80, 23.40)	21.25 (20.20, 22.70)	3.654	0.161
	Healthy side	20.83 (19.85, 22.33)	21.70 (20.60, 23.20)	21.25 (20.20, 22.70)	1.822	0.402
cVEMP amplitude	Affected side	52.05 (30.90, 79.58)	32.90 (19.69, 59.67)	66.42 (43.59, 95.13)	7.917	0.019
(μV)	Healthy side	60.22 (33.30, 96.07)	50.58 (30.33, 70.47)	66.42 (43.59, 95.13)	4.489	0.108
cVEMP-AR		21.33 (12.64, 25.96)	15.06 (4.23, 41.04)	11.49 (5.47, 17.69)	4.127	0.127
N10 (ms)	Affected side	11.09 (10.40, 12.35)	10.50 (10.20, 11.40)	10.8 (10.35, 11.45)	8.529	0.014
	Healthy side	11.17 (10.40, 1.50)	10.70 (10.45, 11.10)	10.8 (10.35, 11.45)	1.433	0.488
P15 (ms)	Affected side	16.15 (14.83, 17.33)	14.80 (14.10, 16.50)	15.55 (14.65, 16.80)	2.223	0.329
	Healthy side	16.40 (15.48, 17.43)	15.50 (14.36, 16.66)	15.55 (14.65, 16.80)	1.822	0.402
oVEMP amplitude	Affected side	5.37 (4.13, 7.66)	5.84 (3.90, 10.34)	8.16 (4.87, 11.85)	3.752	0.153
(μV)	Healthy side	6.61 (3.78, 9.14)	4.50 (3.51, 7.56)	8.16 (4.87, 11.85)	5.810	0.055
oVEMP-AR		13.65 (5.13, 27.20)	19.54 (11.48, 33.18)	13.97 (5.08, 20.09)	2.487	0.288

The amplitude of cVEMP on the affected side in the cp-cu group was 32.90 (19.69, 59.67) μV, while the average amplitude in the control group was 66.42 (43.59, 95.13) μV, with a statistically significant difference (standard test statistic = −2.812, Bonferroni correction *p* = 0.015). The N10 latency of the affected side in the pc-ca group was 11.09 (10.40, 12.35) ms, while that in the control group was 10.8 (10.35, 11.45) ms. The difference was statistically significant (standard test statistic = 2.867, Bonferroni correction *p* = 0.012).

**Figure 1:**
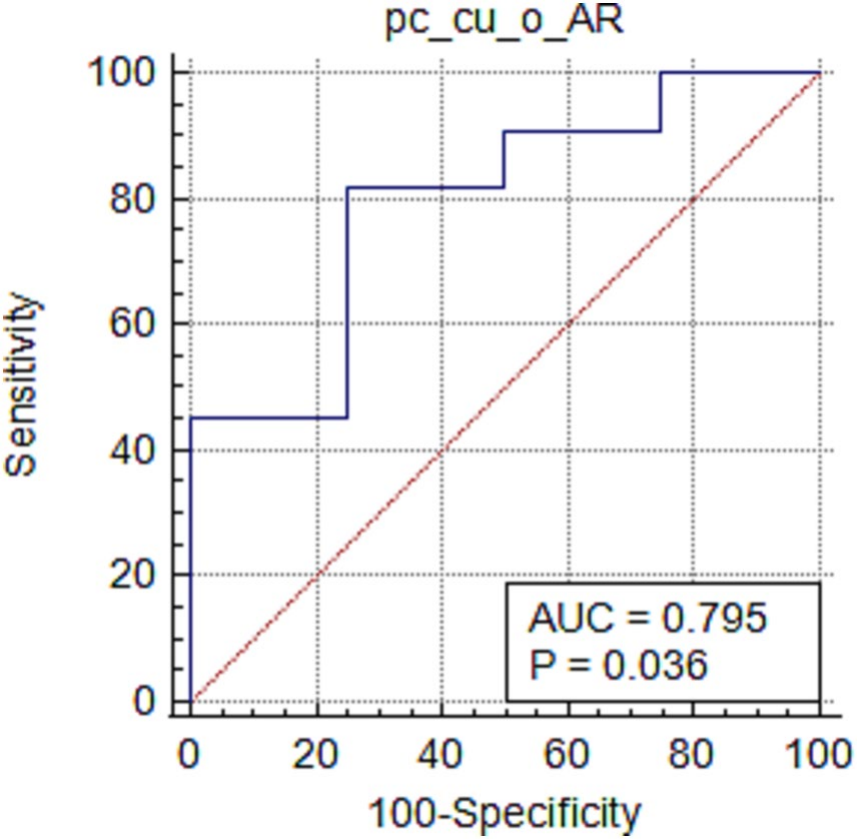
ROC curve of oVEMP-AR and BPPV side in pc-cu group.

The oVEMP-AR in the pc-cu group was correlated with the BPPV side (*Z* = 2.102, *p* = 0.036, Figure 1). The VEMP latency, amplitude, cVEMP-AR in the two groups and the oVEMP-AR in the pc-ca group did not show any correlation with the BPPV side (all *p* > 0.05).

#### Intragroup comparison

The VEMP latency and amplitude of the healthy/affected side in the pc-ca group and the pc-cu group were compared. In the pc-ca group, the cVEMP amplitude of the affected side was 52.05 (30.90, 79.58) μV, and the amplitude of the healthy side was 60.22 (33.30, 96.07) μV, with a statistically significant difference (Mann–Whitney *U* test, *Z* = 2.345, *p* = 0.019); in the pc-cu group, the oVEMP amplitude of the affected side was 5.84 (3.90, 10.34) μV, and the amplitude of the healthy side was 4.50 (3.51, 7.56) μV, with a statistically significant difference (Mann–Whitney U test, *Z* = 3.156, *p* = 0.000). There was no statistically significant difference in the other intra-group comparisons.

### Correlation between vHIT and VEMP abnormalities

A total of 32 cases of pc-ca and 20 cases of pc-cu completed vHIT and VEMP examinations simultaneously. Among them, 14 (43.8%) cases in the pc-ca group had both abnormal results, only 1 (7.1%) case had a close relationship between vHIT and VEMP results (abnormal horizontal semicircular vHIT with ipsilateral oVEMP abnormality), 10 (71.4%) cases contained one bilateral abnormal result, and 3 (21.4%) cases had the opposite side of vHIT and VEMP abnormality. In the pc-cu group, 7 (35.0%) cases had both abnormal results, of which 5/7 (71.4%) cases contained at least one bilateral abnormal result, and 2/7 (28.6%) cases had opposite sides of the two examination results. There was no correlation between vHIT and VEMP abnormality side in both groups (both *p* > 0.05).

## Discussion

The nystagmus components induced by pc-cu in the position test were the same as those induced by pc-ca, that is, the nystagmus is a combination of torsional nystagmus with the upper pole of the eyes beating toward the lower affected ear combined with vertical nystagmus beating upward (toward the forehead). In the HH position, the PC ampulla was most affected by gravity, and the nystagmus was stronger. In the reverse position (i.e., ND position), a weak reverse nystagmus was induced. Due to the heavy cupula mechanism, the duration of nystagmus was more than 1 min, which was different from the typical pc-ca (2, 3).

vHIT is used to detect the high-frequency VOR function of the semicircular canal (10). In this study, both groups of BPPV showed partial vHIT abnormalities, lacking correlation with responsible semicircular canals, and there was no difference between groups. This is similar to previous research results (11, 12). Idiopathic BPPV can be seen with scattered semicircular canal injury, and ectopic otolith is not the main cause of its injury. There was no difference in the abnormal rate of vHIT between the subgroups before/after reduction in the pc-ca group, suggesting that the presence of free otoliths was not the main cause of abnormal vHIT. However, the detachment and mobility of otoliths may have homology with semicircular canal injury (13). However, other studies have suggested that the presence of free otoliths can affect the results of vHIT (4, 14, 15). In this study, most of the abnormal vHITs of BPPV were normal gain with saccades, which can be considered as a relatively light degree of semicircular canal injury (8, 16, 17). Comparing the proportion of saccades between groups, only the proportion of HC saccades in the pc-ca group was higher than that in the control group, and there was no difference between the healthy side and the affected side within the group, which also suggested that there is a lack of correlation between abnormal vHIT and the responsible semi-regulation of bppv. There are few similar reports on pc-cu in previous studies, and the comparative studies on canalolithiasis and cupulolithiasis of the horizontal semicircular canal have different results. Chen (18) reported that there was no statistically significant difference in vHIT, head-shaking test, and temperature test between horizontal semicircular canalolithiasis and cupulolithiasis; Kim et al. (19) reported that that the abnormality of caloric test in horizontal semicircular canalolithiasis was more than that in cupulolithiasis.

Both pc-ca and pc-cu had partial abnormalities in VEMP, but there was no statistically significant difference between the groups. It is noteworthy that although AR, amplitude, and latency comparisons showed some abnormalities on the affected side, these comparisons did not include cases of absence; while in the comparison of abnormal judgments including cases with absence of VEMPs, the abnormal results did not show any association with affected side.

The oVEMP originates from the Utricle, and its conduction pathway is connected with the VOR pathway of AC and HC through the superior vestibular nerve; the cVEMP originates from the Saccule, and its conduction pathway is connected with the VOR pathway of PC through the inferior vestibular nerve (20, 21). There was no correlation between the location of VEMP lesions and vHIT, suggesting that these lesions were scattered and probably mainly from the vestibular peripheral organs.

It is generally believed that BPPV is more closely related to the Utricle and oVEMP (5, 22). However, the causes of Utricle degeneration and injury are usually systemic, and Saccule function is often involved at the same time (23, 24), and is not limited to the injured side (25). The temporal bone study found that the saccule and subvestibular nerve cell counts were reduced in the ear of BPPV, suggesting that BPPV may be related to the injury of saccule and subvestibular nerves. The study of temporal bone found that the cell count of saccule and inferior branch of vestibular nerve in BPPV ear was reduced, suggesting that BPPV may be related to the injury of saccule and inferior vestibular nerve (26, 27); however, some researchers believed that it may be caused by aging (5).

Manual reduction therapy cannot cure pc-cu (28, 29). The vast majority of patients with pc-cu do not transform into typical pc-ca in the process of self-healing, which may be related to the adhesion of otolith particles on the short arm side of the cupula (30), but it cannot be excluded that it may be related to other mechanisms that lead to heavy cupula of PC. Kim et al. (9) proposed that the mechanism of apogeotropic nystagmus may be related to otolith organ injury, and believed that this may be the reason why cupulolithiasis is difficult to convert into canalolithiasis.

In summary, the vHIT and VEMP results of the pc-ca group and the pc-cu group were partially abnormal, mostly lacking correlation with the semicircular canals responsible for BPPV, suggesting that both groups had scattered vestibular peripheral organ injury; the injury was not caused by BPPV, but may have homology with the pathogenesis of BPPV. There was no significant difference in the vHIT and VEMP results of the two groups of BPPV.

### Deficiencies and prospects

vHIT and VEMP are not necessary examinations for the clinical diagnosis and treatment of typical BPPV. Due to the limitations of retrospective studies, although this study has tried to exclude possible secondary BPPV, some BPPV cases with simple medical history and clear diagnosis were not included in the study due to the lack of vHIT examination, which may lead to a high positive rate of the two vestibular function tests in this study. In addition, age and aging can affect the results of VEMP (31). The small sample size of VEMP in this study cannot consider age-related factors more, which may lead to a high positive rate of VEMP.

## Data availability statement

The datasets presented in this article are not readily available because of ethical and privacy restrictions. Requests to access the datasets should be directed to XW, 181934096@qq.com.

## Ethics statement

The studies involving humans were approved by Ethics Committee of Dongzhimen Hospital Affiliated to Beijing University of Chinese Medicine. The studies were conducted in accordance with the local legislation and institutional requirements. The ethics committee/institutional review board waived the requirement of written informed consent for participation from the participants or the participants’ legal guardians/next of kin because this study was a retrospective analysis, and the risk to the subjects was less than the minimal risk.

## Author contributions

XW: Writing – original draft, Writing – review & editing. YL: Writing – review & editing. WJ: Writing – review & editing. JH: Writing – review & editing.
